# Prevalence and associated risk factors of tinnitus among adult Palestinians: a cross-sectional study

**DOI:** 10.1038/s41598-022-24015-w

**Published:** 2022-11-30

**Authors:** Saad Al-Lahham, Zaher Nazzal, Ahmad Massarweh, Diana Saymeh, Sojoud Al-abed, Donia Muhammad, Raghda Alawni, Noor Bouzya, Malik Alqub, Mustafa Ghanim, Nael Ellahham

**Affiliations:** 1 Department of Biomedical Sciences, Faculty of Medicine and Health Sciences, An-Najah National University, P.O. Box 7, Nablus, Palestine; 2 Department of Medicine, Faculty of Medicine and Health Sciences, An-Najah National University, P.O. Box 7, Nablus, Palestine; 3 Department of Pediatric Cardiology, Almakassed Charitable Hospital, Jerusalem, Palestine

**Keywords:** Risk factors, Diseases

## Abstract

Tinnitus is a common complaint with potentially negative impact on quality of life. Prevalence of tinnitus ranges from 5 to 43% worldwide. This variation could be due to the heterogeneity of tinnitus assessment. This has limited the progress in understanding tinnitus. Therefore, we employed a standardized and a validated assessment method to determine the prevalence and related risk factors of tinnitus among Palestinians for the first time. This is a cross-sectional study in which we questioned a representative sample of 618 subjects in one-to-one interviews. The prevalence of any tinnitus was 30.6% among adult Palestinians. Participants from the oldest age group were almost five times more likely to have tinnitus. Moreover, participants with head and neck pain syndrome, severe hearing impairment, sleeping disorders or frequent complaints of vertigo were approximately two times more likely to have tinnitus. Our study provides novel information regarding tinnitus in Palestine and improves our understanding of tinnitus. This will improve the diagnosis and consequently will contribute in reducing the prevalence and perhaps in preventing tinnitus. As tinnitus still has no known cure, further investigations of modifiable risk factors and causes of tinnitus are crucial to prevent it in the future.

## Introduction

Tinnitus is the perception of sound with no corresponding external acoustic stimulation^1^. It is mainly classified into either subjective or objective tinnitus. The subjective tinnitus is perceived only by the patient and it is the most common form^2^. On the other hand, the objective tinnitus can be audible to an examiner and perceived by the patient^3^. Tinnitus is not a disease by itself, but rather a symptom associated with a variety of diseases and risk factors. Tinnitus severity ranges from slight forms that patients hear only in quiet environments and has no impact on their life, to severe or even catastrophic forms that patients always hear and are associated with disturbed sleep patterns and difficulties performing daily activities^4^. Furthermore, previous studies have shown an association between tinnitus and various psychological problems including depression and anxiety^5,6^. Treatment of tinnitus can be costly and places a considerable financial burden on public health care systems. For example, the annual cost of tinnitus treatment in the United Kingdom was estimated to be GB£750 million^7^. Therefore, determining the prevalence of tinnitus and the frequency of its risk factors is important to improve diagnosis and prevention.

The global prevalence of tinnitus ranges from 5 to 43%^8^. Similarly, the prevalence rates of tinnitus reported in some Middle Eastern countries also vary. For example, a study from Turkey reported a high prevalence of tinnitus (33%), while another study from Iran showed a relatively low prevalence (4.6%)^9,10^. The definitions of tinnitus and the survey questions that were utilized to determine the prevalence in these studies were not clearly stated, which makes it difficult to determine whether differences in these parameters have contributed to the variations in the results. Nonetheless, it is evident that the sampling methods in these studies are different and this may have contributed to the variations in results. In general, variations in the prevalence of tinnitus may arise from the heterogeneity of tinnitus assessment, tinnitus criteria^8^, associated risk factors and underlying mechanisms and clinical manifestations^11^. Indeed this has been shown by a recent review of 86 studies, in which 42 discrete complaints were recognized. In addition, several tinnitus profiling questionnaires have been proposed and none of them could cover all of these diverse aspects. However, The European School for Interdisciplinary Tinnitus Research Screening Questionnaire (ESIT-SQ), which was recently developed and validated, overcomes this issue^12^. ESIT-SQ addresses the heterogeneous aspects of tinnitus by including all questions present in The Tinnitus Sample Case History Questionnaire (TSCHQ), the mainstay of sub-typing analyses using The Tinnitus Research Initiative, but in greater detail and it includes the vast majority of domains found in other instruments and earlier studies. TSCHQ includes only a few questions about co-existing medical conditions. Therefore, it is not suitable for acquiring information about the tinnitus-relevant medical history such as the presence of otological or tinnitus-related comorbid systemic diseases, the history of medical treatments or the history of medical procedures that can cause tinnitus^12^. Furthermore, ESIT-SQ is designed to collect data from the entire adult population, tinnitus and non-tinnitus, which are important to study the mechanisms associated with tinnitus and this is a unique criteria of this instrument. Finally, it combines clinical expertise and analysis of big datasets at the same time to determine the related risk factors and therefore it contributes to a wider understanding of tinnitus. To our knowledge, no study has addressed the topic of tinnitus in Palestine and therefore, we conducted this study.

## Results

### Background characteristics of the participants

A total of 618 participants [284 (46.0%) males and 334 (54.0%) females] were included in this study. Their age range was between 20 and 80 years old with the majority of them under 40 years old (66%) and a minor fraction was more than 60 years old (6%). Almost half of the participants lived in cities (46.7%) and the other half lived in villages (53.3%). More than half of the participants were overweight or obese (55.9%). The majority of the participants were right-handed (83.3%) and 62.6% of the participants had an educational level above secondary school. Current smoking was reported in 204 (33.0%) participants and 105 (17.0%) had a family history of tinnitus. Table 1 presents in detail the background characteristics of the study participants.

**Table 1:** Background Characteristics of the study participants (n = 618).

	Frequency	Percentage (%)
**Sex**		
Male	284	46
Female	334	54
**Age**		
< 40 years	408	66
40–59 years	197	29
≥ 60 years	31	6
**BMI**		
Underweight (< 18.5)	11	1.80
Normal weight (18.5–24.9)	262	42.40
Overweight (25–29.9)	221	35.80
Obesity (≥ 30)	124	20.10
**Handedness**		
Right	515	83.30
Left	52	8.40
Bilateral	51	8.30
**Residency**		
City	288	46.60
Villages	330	53.40
**Telephone use**		
Yes—frequently	396	64.10
Yes—rare	105	17.30
No	117	18.90
**Educational level**		
Up to secondary school level	231	37.40
Above secondary school level	387	62.60
**Smoking**		
Never smoke	373	60.40
Ex-smoker	39	6.30
Current smoker	204	33.00
**Family history of tinnitus**		
Yes	105	17.00
No	513	83.00

### Clinical characteristics of the participants

As demonstrated in Table 2, the survey revealed that 50.6% of the participants had a history of vertigo. The survey also indicated that 39.9% of the participants had hearing problems and 33.7% of the participants reported being annoyed by external sounds, while only 7.8% had a history of ear, head and neck operations. In addition, it revealed that 50.3%, 14.1%, 11.3%, 7%, 11.3%, 15% and 17.2% of participants had head and neck pain syndrome, sleeping disorders, cardiovascular diseases, balance disorders, endocrine and metabolic diseases, depressive symptoms and otorhinolaryngological problem, respectively (Table 2).

**Table 2:** Clinical characteristics of the study participants (n = 618).

**Vertigo**		
Never	305	49.40%
Yes (< 1episode per year)	136	22.00%
Yes (≥ 1 episode per year)	177	28.60%
**History of ear, head and Neck operations**		
Yes	48	7.80%
No	570	92.20%
**Annoyed by external sounds**		
Yes	208	33.70%
No	410	66.30%
**Hearing problems**		
Yes, cannot hear at all	62	10.00%
Yes, slight difficulty	185	29.90%
No difficulty	371	60.00%
**Head and neck pain syndrome**		
Yes	311	50.30%
No	307	49.70%
**Depressive and anxiety symptoms**		
Yes	93	15.00%
No	525	85.00%
**Sleeping disorder**		
Yes	87	14.10%
No	531	85.90%
**Cardiovascular diseases**		
Yes	70	11.30%
No	548	88.70%
**Endocrine and metabolic disease**		
Yes	70	11.30%
No	548	88.70%
**Otorhinolaryngological problem**		
Yes	106	17.20%
No	512	82.80%
**Balance disorders**		
Yes	43	7.00%
No	575	93.00%

### Prevalence of tinnitus and its characteristics

**Figure 1:**
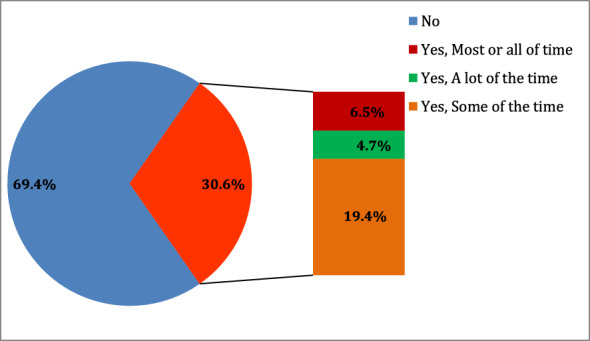
Prevalence of tinnitus. Prevalence of tinnitus among the studied sample and the frequency of tinnitus over the past year (n = 618).

As shown in Fig. 1, the overall prevalence of tinnitus among our population was 30.6% (n = 189) [95% CI: 27.0–34.4%]. More than one-third of them had it either most or a lot of the time (Fig. 1), while the remaining 19% suffered from tinnitus in only some of their time. Table 3 describes the features of tinnitus among participants who reported to have it. Tinnitus affected 28.6% of the participants on a daily or almost-daily basis. 59.9% of the affected participants perceived tinnitus bilaterally with more than half of them describing equal intensity in both ears. Tinnitus was constant among 49.2% of the affected participants and sounded like noise in 48.1% of the affected participants. The majority of tinnitus cases (67.7%) followed no rhythm. At least 4.8% of the participants confirmed objective tinnitus. As shown in Table 4, exposure to loud sounds was perceived to be related to the onset of tinnitus among 22.2% of the affected participants. 24.9% of those with tinnitus stated that they consumed painkillers around the time of onset of tinnitus. The majority of cases sought no management for tinnitus and only 2.6% of the participants reported that they used medications as a management option. Further characteristics of tinnitus onset and management options that the affected participants underwent are listed in Table 4.

**Table 3:** Characteristics of tinnitus among the affected participants (n = 189).

	Frequency	Percentage (%)
**Frequency of tinnitus (n = 189)**		
Daily or almost daily	54	28.60
Almost weekly	44	23.30
Almost monthly	28	14.80
Every few months	46	24.30
Yearly	17	9.00
**Description of tinnitus**		
Constant	93	49.20
Intermittent	96	50.80
**Tinnitus sound like**		
Noise	91	48.10
Crickets	41	21.70
Tonal	40	21.20
Music	8	4.20
Other	9	4.8
**Tinnitus rhythm**		
No rhythm	128	67.70
Yes, follow heart beat	20	10.60
Yes, follow head, neck, or jaw movements	20	10.60
Yes, following breathing	15	7.90
Other	6	3.20
**Place where tinnitus s perceived**		
Both ears, equally	63	33.30
Right ear	36	19.00
Both ears, worse in right	29	15.30
Both ears, worse in left	21	11.30
Left ear	17	9.00
Inside the head	12	6.30
Do not know	11	5.80
**Heard by clinician**		
Yes	9	4.80
No	180	95.20

**Table 4:** Characteristics of Tinnitus onset and management options among study participants (n = 189).

	Frequency	Percentage (%)
**Onset of tinnitus is related to**		
Exposure to loud sounds	42	22.20
Change in hearing	23	12.10
Flu or common cold	16	8.40
Change in ambient pressure	8	4.20
Feeling of fullness or pressure in the ears	8	4.20
Stress	10	5.20
Head trauma	6	3.10
Neck trauma	6	3.10
Others	8	4.20
None	62	32.80
**Medicines consumed around the time of tinnitus onset**		
Pain killing medication	47	24.90
Aspirin	24	12.70
Antibiotics	24	12.70
Diuretics	11	5.80
Oral steroids	7	3.70
Other medicines	28	14.80
No medication	96	50.80
**Seen a healthcare professional**		
Yes, 5 or more visits	15	7.90
Yes, from 2 to 4 visits	26	13.80
Yes, just one visit	19	10.10
Not at all	129	68.30
**Management**		
Self-management (e.g. dietary supplements, support groups, relaxation)	14	7.40
Medications	5	2.60
Others (psychological management, audiological management and physiotherapy)	9	4.70
No management	161	85.20

### Factors associated with tinnitus

Univariate and multivariate analyses were conducted to assess factors associated with tinnitus among the study’s participants. All relevant variables were included in multivariate logistic regression to predict the relation with tinnitus and control for confounders (Table 5). Gender, obesity (BMI ≥ 30), place of residence, telephone use, smoking, family history of tinnitus, use of hearing aids, cardiovascular diseases and endocrine and metabolic diseases were not significantly associated with tinnitus. On the other hand, it appeared that older age, hearing problems, head and neck pain syndromes (including pain from ears, face, and the temporomandibular joint), sleeping disorders, and vertigo were more frequently and significantly associated with tinnitus. The oldest age group (≥ 60 years) was 4.8 times more likely to have tinnitus compared to the youngest age group (< 40 years*) *[*AOR* = 4.8, *95% CI* *= 1.8–12.3*]. Participants with severe hearing problems were 2.2times more likely to have tinnitus compared to those without hearing problems [*AOR* = 2.2, *95% CI* = 1.1–4.3]. In addition, head and neck pain syndrome and sleeping disorder were associated with more frequency of tinnitus, [*AOR* = 2.2, *95% CI* = 1.4–3.4] and [*AOR* = 2.1,* 95% CI* = 1.2–3.6], respectively. Lastly, participants with frequent complaints of vertigo (≥ one episode per year) were almost 2 times more likely to have tinnitus [*AOR* = 1.9,* 95% CI* = 1.2–3.0].

**Table 5:** Analysis of potentially associated factors with tinnitus.

Characteristics	Tinnitus yes (n = 189)	Tinnitus no (n = 429)	Univariate analysis: crude OR (95%CI)	Multivariate analysis	
	Frequency (%)	Frequency (%)		Adjusted OR	95% CI
**Sex**					
Male*	72 (25.4)	212 (74.6)	1	1.1	0.73–1.7
Female	117 (35.0)	217 (65.0)	1.6 (1.1–2.3)		
**Age**					
< 40 years*	104 (25.5)	304 (74.5)	1		
40–59 years	66 (36.9)	113 (63.1)	4.6 (2.2–9.8)	2.6	1.1–6.6
≥ 60 years	19 (61.3)	12 (38.7)	2.7 (1.2–5.9)	**4.8**	**1.8–12.6**
**Obesity (BMI ≥ 30)**					
Yes	49 (39.5)	75 (60.5)	1.7- (1.1–2.5)	1.3	0.75–2.1
No*	140 (28.3)	354 (71.7)	1		
**Residency**					
City*	81 (28.1)	207 (71.9)	1	1	
Villages	108 (32.7)	222 (67.3)	1.2 (.88–1.8)	1.02	0.67–1.6
**Telephone use**					
Yes—frequently	116 (29.3)	280 (70.7)	1.1 (0.68–1.7)	1.4	0.74–2.5
Yes—rare	37 (35.2)	68 (64.8)	0.82 (0.47–1.4)	1.4	0.80–2.4
No	36 (30.8)	81 (69.2)	1	1	
**Educational level**					
Up to secondary school level*	83 (35.9)	148 (64.1%0	1.5 (1.1–2.1)	1.1	0.71–2.6
Above secondary school level	106 (27.4)	281 (72.6)	1	1	
**Smoking**					
Never smoke*	123 (33.0)	250 (67.0)	1	1	
Ex-smoker	13 (33.3)	26 (66.7)	0.98 (.49–2.0)	1.1	0.45–2.6
Current smoker	53 (25.7)	153 (74.3)	1.4 (.97–2.1)	1.5	0.81–2.4
**Family history of tinnitus**					
Yes	44 (41.9)	61 (58.1)	1.8 (1.2–2.8)	1.6	0.93–2.6
No*	145 (28.3)	368 (71.7)	1		
**Vertigo**					
Never*	64 (21.0)	241 (79.0)	1	1	
Yes (< 1 episode per year)	47 (34.6)	89 (65.4)	3.0 (2.0–4.5)	1.5	0.89–2.6
Yes (≥ 1 episode per year)	78 (44.1)	99(55.9)	1.5 (0.94–2.4)	**1.9**	**1.2–3.0**
**Balance disorders**					
Yes	30 (15.9)	159 (84.1)	6.0 (3.1–11.9)	2	0.92–4.4
No*	13 (3.0)	416 (97.0)			
**Annoyed by external sounds**					
Yes	84 (40.4)	124 (59.6)	2.0 (1.4–2.8)	1.2	0.78–1.8
No*	105 (25.6)	305 (74.4)			
**History of ear, head and neck operations**					
Yes	21 (43.8)	27 (56.3)	1.9 (1.1–3.4)	1.2	0.56–2.4
No*	168 (29.5)	402 (70.5)	1		
**Hearing problems**					
Yes, cannot hear at all	31 (50.0)	31 (50.0)	4.1 (2.3–7.1)	**2.2**	**1.1–4.3**
Yes, slight difficulty	85 (45.9)	100 (54.1)	1.2 (0.66–2.1)	0.86	0.43–1.8
No difficulty*	73 (19.7)	298 (80.3)	1		1
**Depressive and anxiety symptoms**					
Yes	44 (47.3)	49 (52.7)	2.4 (1.5–3.7)	1.6	0.90–2.8
No*	145 (27.6)	380 (72.4)	1		
**Using hearing aids**					
Yes	9 (47.4)	10 (52.6)	2.1 (.83–5.2)	1.1	0.32–3.3
No*	180 (30.1)	419 (69.4)	1		
**Head and neck pain syndromes**					
Yes	136 (43.7)	175 (56.3)	3.7 (2.6–5.4)	**2.2**	**1.4–3.4**
No*	53 (17.3)	254 (82.7)	1		
**Sleeping disorder**					
Yes	46 (52.9)	41 (47.1)	3.1 (1.9–4.8)	**2.1**	**1.2–3.6**
No*	143 (26.9)	388 (73.1)	1		
**Cardiovascular diseases**					
Yes	37 (52.9)	33 (47.1)	2.9 (1.8–4.8)	1.2	0.65–2.4
No*	152 (27.7)	396 (72.3)	1		
**Endocrine and metabolic disease**					
Yes	33 (47.1)	37 (52.9)	2.2 (1.4–3.7)	1.3	0.69–2.5
No*	156 (28.5)	392 (71.5)	1		
**Otorhinolaryngological problem**					
Yes	51 (48.1)	55 (51.9)	2.5 (1.6–3.9)	1.6	0.96–2.6
No*	138 (27.0)	374 (73.0)	1		

*AOR* adjusted odds ratio, *CI* confidence interval*.*

Significant values are in bold.

*Reference group.

## Discussion

Tinnitus is a highly prevalent condition with a global prevalence that ranges from 5 to 43%^8^. Several risk factors and disease conditions are associated with tinnitus^13^. Information regarding the prevalence and the nature of the risk factors associated with tinnitus in Palestine is lacking. This is the first national study providing representative data on tinnitus prevalence in the Palestinian population. In the current study, we found that the overall prevalence of any tinnitus in Palestine was 30.6%. This is similar to the prevalence in Australia (30.3%) and Turkey (33%), a neighboring Middle Eastern country^9,14^. Yet, it is higher than the prevalence rates reported in other Middle Eastern countries such as Iran (4.6%) and Egypt (5.2%)^10,15^. Also, it is two times higher than the prevalence observed in the USA (14.3%)^16^. These variations in the prevalence between countries could be due to real intrinsic differences in tinnitus occurrence and management between populations resulting from differences in the social, cultural, economic and environmental conditions as well as the individual’s biological traits and behavior which influence the population health^17^. It is possible that limited accessibility to health care or the stigmatization of mental-health difficulties associated with tinnitus, which may restrain individuals from receiving the best diagnostic/therapeutic services, can also contribute to the differences in prevalence rates of tinnitus between the different communities^18–20^. Variations could also be due to the heterogeneity in the setup of the studies as well as the lack of a standard and validated definition of tinnitus. This makes comparisons between different surveys hard. Therefore, we employed a recent standardized and validated assessment method, i.e. ESIT-SQ^12^.

Hearing loss, whether noise-induced or age-related (i.e. presbycusis), is one of the major etiologic factors associated with tinnitus that originates within the auditory pathways^13,21,22^. Age-related hearing loss and associated tinnitus may explain the tendency of tinnitus prevalence to increase with age, a phenomenon reported in several previous studies^16,23–26^. In our study, tinnitus prevalence increased from 25.5% in participants < 40 years old to 36.9% in participants 40–59 years old and 61.3% in participants ≥ 60 years old. Moreover, multivariate analysis indicated that participants in the oldest age group (≥ 60 years old), were almost five times more likely to have tinnitus compared to the youngest age group. In addition, we found that the participants with self-reported severe hearing problems were two times more likely to have tinnitus compared to those without hearing problems. These results are similar to the findings by McCormack et al*.* and Khedr et al*.*^15,23^.

Somatosensory tinnitus is the form of tinnitus generated or affected by inputs from somatosensory, somatomotor or visual-motor systems^27^. Tinnitus associated with disorders of temporomandibular joint and other somatic disorders of the head and neck as well as pain syndromes originating from head and neck structures is considered somatosensory tinnitus^28–32^. In our study, the prevalence of tinnitus in participants with a positive history of head and neck pain syndromes was 43.7% compared to only 17.3% in those without similar history. In addition, multivariate analysis revealed that head and neck pain syndrome among participants with tinnitus was two times more common compared to those without pain syndrome.

Several disorders are known to present with tinnitus and vertigo together especially disorders that involve inner ear, vestibulocochlear nerve, or the central nervous system pathways connected to the vestibular or auditory systems^33^. Examples of such disorders include; Ménière's disease, vestibular schwannoma, atmospheric inner ear barotrauma, vertebrobasilar ischemic stroke and otosclerosis^34,35^. A report by Ren Y.F. and Isberg A., showed that vertigo was nearly four times more common in patients with tinnitus than in those without vertigo^36^. Our study revealed that the prevalence of tinnitus in participants with more than one episode of vertigo per year (44.1%) was significantly two times higher than the prevalence of tinnitus in participants who never had tinnitus (21%).

Association between tinnitus and sleeping disorders has been described previously^37,38^, but the cause–effect relationship between sleeping disorders and tinnitus is difficult to confirm. This is because of the possibility that sleep disorders may be a complication of tinnitus itself as well as the possibility of both arising from a common pathology^39^. Our present study shows that the prevalence of tinnitus in subjects with sleeping disorders was significantly two times more common compared to those without sleep disorders (52.9% vs. 26.9%), which is in line with the previously mentioned studies^37,38^.

Our results revealed that tinnitus was more prevalent in females (35%) than in males (25.4%), which is similar to the findings in a Turkish study that investigated tinnitus in the visitors of primary care centers^9^. Increased prevalence of tinnitus and tinnitus annoyance in females was also shown in a survey on the population of South Korea by Park et al. Those authors attributed their findings to the more stressful cultural situation of South Korean women that demands female obedience and more roles of women within the family^40^. Palestinian women suffer similar cultural stressors in addition to political violence arising from the military occupation^41^, which may have contributed to the increased perception of tinnitus in women in our study. On the other hand, it is possible that the decreased prevalence of tinnitus in Palestinian males arises from the tendency of males to not declare their health problems^42^. Nonetheless, our multivariate analysis revealed no significant association between gender and tinnitus complaint. It should be noted that prevalence of tinnitus in different sexes has been investigated in several other studies with diverse results^8,23,24,40,43^. The majority of these studies revealed that tinnitus is more common in men than in women, which may reflect the reality that men are more involved than females in occupations and leisure activities that expose them to noise and noise-induced hearing loss^44,45^.

Previous studies indicated increased prevalence of psychiatric disorders in patients with tinnitus compared controls. For example, Sullivan et al. reported a lifetime major depression prevalence rate of 78% in patients with tinnitus compared to only 21% in controls (60% vs. 7% for current depression)^5^. In addition, a study by Bhatt et al., showed that 25.1% of participants who reported to have tinnitus in the past year also suffered depressive problems in the same period compared to only 9.1% of participants with depression symptoms who report to have no tinnitus. The same study reported similar results for anxiety related problems^6^. A recent study by Kao et al., revealed that major depressive disorder may be one of the risk factors for tinnitus onset^46^. Another study by Hou et al., revealed a significant increase in the lifetime incidence of tinnitus in patients with anxiety disorder^47^. In our survey, we found that 47.4% of tinnitus-affected participants reported depressive and anxiety symptoms compared to only 27.6% of those tinnitus-affected participants who reported no such symptoms, while our multivariate analysis revealed no significant association between this variable and tinnitus. It is worth noting that the prevalence of loneliness, anxiety and depressive disorders increases with age^48^. This may have some contribution to the increased prevalence, severity of tinnitus and negative impact on the quality of life in the elderly population. Studies that address these associations in the elderly comprehensively are scarce^49^.

A study by Kim et al. reported a significant association between having a family history of tinnitus and tinnitus in adolescents^50^. In addition, a multinational European study showed that tinnitus aggregates in families^51^. In the light of these reports, we expected a similar familial effect on tinnitus in our sample. Our study shows that 41.9% of tinnitus-affected participants have a family history of tinnitus compared to only 28.3% of those tinnitus-affected participants without family history. Nonetheless, we could not identify a significant association between family history of tinnitus and tinnitus complaints in multivariate analysis.

Otorhinolaryngological conditions that cause Eustachian tube dysfunction are known to cause tinnitus^52,53^. A study by Manche et al., reported a tinnitus prevalence of 29.3% in patients presenting to an Ear, Nose, and Throat (ENT) hospital with various otological diseases^54^. On the one hand, our present study shows that the prevalence of tinnitus in subjects with otorhinolaryngological problems is 48.1% compared to only 27% in subjects without otorhinolaryngological problems, which is even higher than prevalence rate reported in the aforementioned study by Manche et al. Multivariate analysis on the other hand revealed no significant association between tinnitus and otorhinolaryngological problems.

Ear, head or neck operations may rarely be complicated with iatrogenic pulsatile tinnitus^55–57^. A small number of our study participants reported a positive history of ear, head or neck operations and the prevalence of tinnitus in those participants was 43.8% compared to only 29.5% in those with negative history. Multivariate analysis could not identify a significant association between having a history of such operations and tinnitus. The small number of participants with this risk factor is probably insufficient for exploring the association with tinnitus.

Finally, the high prevalence rate of tinnitus and the nature of the most prevalent tinnitus associations revealed in our study population, especially advanced age and hearing difficulties, demand for multimodal management approaches that address both hearing-related and mental health difficulties. In particular, management options for patients with chronic distressing tinnitus that is associated with hearing difficulties include hearing aids and/or cognitive behavioral therapy as recommended by currently available treatment guidelines^58,59^.

This study provides novel information regarding tinnitus in Palestine and improves our understanding of tinnitus. However, the study still has some limitations to be considered. First, this cross-sectional study addresses tinnitus in the Palestinian population at a specific time point and gives no information about its incidence. Furthermore, it is not possible to conclude a cause–effect relationship between a risk factor and the occurrence of tinnitus using such a study design. Second, while we believe that a sample size of 618 is adequate to define the prevalence of tinnitus and some of its characteristics; it may be insufficient for exploring the associations with specific risk factors. Third, employing ESIT-SQ instrument, we investigated and profiled tinnitus risk factors separately. Therefore, we aim in the future to investigate these risk factors in a wider model such as biopsychosocial model to further understand tinnitus and the underlying mechanisms and improve its diagnosis. Finally, the current study investigated the characteristics and the potential risk factors of tinnitus in Palestinian adults and did not address this disorder in adolescents and children. Therefore, further work is required to establish the incidence of tinnitus in the Palestinian population and to investigate the cause–effect relationship for frequent risk factors associated with tinnitus in our population. Further research regarding tinnitus characteristics and risk factors in Palestinian younger age groups is also required; perhaps this will prevent it at an early stage.

In conclusion, our findings reveal that tinnitus is prevalent among Palestinians. Our study demonstrates that advanced age, hearing difficulties, head and neck pain syndromes, vertigo, sleeping disorders, and otorhinolaryngological problems are among the most frequent factors associated with tinnitus. As there is no known definitive cure for many forms of tinnitus, it is crucial to investigate potentially modifiable risk factors and subsequently prevent tinnitus. Therefore, further longitudinal (repeat cross-sectional or prospective) studies are required.

## Materials and methods

### Study design and study population

This study is a cross-sectional study that was conducted in the West Bank of Palestine in 2020. The total population of the West Bank is 3,120,448 (2020–2021) (PCBS-2017)^60^. A convenient sampling technique was implemented to select five municipalities to represent the West Bank, i.e., Nablus, Jenin, Salfet, Bethlehem and Hebron. The sample size needed to meet the research objectives with adequate statistical power was calculated using the following equation:

$$n\, = \,[{\text{DEFF}}*{\text{Np}}({1} - {\text{p}})\left] / \right[({\text{d}}^{{2}} /{\text{Z}}^{{2}}_{{{1} - \alpha /{2}}} *({\text{N}} - {1})\, + \,{\text{p}}*({1} - {\text{p}})].$$

The sample size (*n*) was estimated using a population size (*N*) of one million, % frequency of the outcome factor in the population (*p*) = 50%, precision (*d*) = 4%, design effect (DEFF) = 1, and confidence level = 95%. Hence, the minimum adequate total sample size was 600. Participants who were less than 20 years old, who refused to measure their height and weight or who did not answer any of the questions during the interview were excluded. Ultimately, 618 participants (285 male, 335 female) were included in this study. We believe this sample size collected from five municipalities represent the West Bank of Palestine, as it is a small geographical area of about 5600 km^2^ with a population of around three million people. Furthermore, the population is almost similar in all municipalities and the chosen municipalities are among the largest in the West Bank^61^. Finally, our data is reflected in the general Palestinian population, as we observed a similar distribution of gender and age^62^.

### Data collection

The study was approved by An-Najah National University Institutional Review Board (5/January/2020) and conducted according to the Helsinki declaration. Written informed consent was obtained from all participants prior to the survey. Although municipalities were conveniently chosen; participants were randomly selected from the corresponding municipalities, interviewed and examined. The data was collected employing ESIT-SQ, which consists of 39 closed questions structured in two parts.^12^The first part includes 17 questions about demographics, body characteristics, education, life-style, family history, medical and surgical history, presence of hearing-related problems and other factors. All participants regardless of whether they have tinnitus or not can answer this first part. The last question of the first part is phrased as follows: “Over the past year, have you had tinnitus in your head or in one or both ears that lasts for more than five minutes at a time?” This questions screens for the presence of any tinnitus (chronic or acute, monaural or binaural, and constant or intermittent) lasting for more than 5 min over the past year. Overall prevalence rate was calculated from the percentage of all participants who answered “yes” to the question. Participants who answered “yes” to this question were asked to answer 12 more tinnitus-related questions in the second part. These include questions about tinnitus perceptual characteristics, the impact of tinnitus, onset-related characteristics, tinnitus-modulating factors and associations with co-existing conditions, objective tinnitus, and two healthcare-related questions. ESIT-SQ was translated into Arabic with slight modifications. Few questions on medical and tinnitus history were adapted and included to suit the objectives of the study and the population sample. A question related to alcohol abuse was removed because it is not applicable to our community. Some questions were added to study the relation of occupation, and mobile use with tinnitus. Body weight, height and body mass index (BMI) were measured and added to the study. Weight was measured to the nearest 0.1 kg in light clothing using a digital balance. Standing height was measured to the nearest 0.5 cm. BMI was calculated as weight in kilograms divided by height in meters squared. It was categorized based on WHO guidelines. The categories were underweight (< 18.5 kg/m^2^), normal weight (18.5–24.9 kg/m^2^), overweight (25–29.9 kg/m^2^), and obese (> 30 kg/m^2^).

### Statistical analysis

Data was entered and analyzed using the SPSS software. Continuous variables were presented as mean ± standard deviation (SD) and frequency and percentages were used to describe categorical variables using tables and charts, as appropriate. The prevalence and 95% confidence intervals (CIs) of tinnitus were determined. In univariate analysis, Chi-square test was used to determine the relation between tinnitus and risk factors. Additionally, we computed adjusted odds ratios (AORs) with their 95% Confidence Intervals (95% CI) using multivariable analysis. All relevant variables were included in multivariate logistic regression. All P-values were two-sided, and P < 0.05 was considered statistically significant.

## Data Availability

The datasets generated and/or analyzed during the current study are not publicly available due the fact that it contains information, which could compromise the privacy of research participants, but are available from the corresponding author on reasonable request.
